# THE STEP-HI STUDY: IMPACT OF TESTOSTERONE AND RESISTANCE EXERCISE ON SPPB, LEG STRENGTH, AND APPENDICULAR LEAN MASS

**DOI:** 10.1093/geroni/igae098.0272

**Published:** 2024-12-31

**Authors:** Elena Volpi, Douglas Kiel, Rocco Paluch, Peter Dore, Richard Fortinsky, George Kuchel, Denise Orwig, Ellen Binder

**Affiliations:** University of Texas Health San Antonio, San Antonio, Texas, United States; Harvard Medical School, Boston, Massachusetts, United States; University at Buffalo, SUNY, Buffalo, New York, United States; Washington University School of Medicine, St. Louis, Missouri, United States; University of Connecticut, Farmington, Connecticut, United States; University of Connecticut, Farmington, Connecticut, United States; University of Maryland School of Medicine, Baltimore, Maryland, United States; Washington University School of Medicine, St. Louis, Missouri, United States

## Abstract

The STEP-HI Study tested if testosterone replacement therapy in combination with resistance exercise training for 6 months could improve functional recovery from hip fracture in older women more than resistance exercise alone or standard of care. Here we present the results of the intention to treat analyses (N=122) of the effect of testosterone replacement plus exercise (EX+T, n=53) vs. exercise plus placebo (EX+P, n=51) vs. enhanced usual care (EUC, n=18) on the Short Physical Performance Battery (SPPB) scores, maximal leg press strength, and appendicular lean mass. Group differences were analyzed with contrasts using a mixed model repeated analysis of variance that included the following covariates for each outcome: clinical site, age, days since hip surgery, BMI, hip fracture type, general depression score, lung disease, comorbidity index and baseline level. EX+T had a greater improvement in SPPB score than EX+P (p<0.009). Maximal leg press strength increased in all groups by the end of the intervention period with no differences between groups (p>0.05). Appendicular lean mass increased to the same extent in the EX+T and EX+P groups, but slightly decreased in the EUC group as compared to EX+T (p=0.020) and EX+P (p=0.029). These findings suggest that testosterone may independently impact a composite measure of physical performance, while exercise significantly improves appendicular lean mass independent of testosterone replacement. The implications of these findings will be discussed.

